# Potential role of polydatin in treating diabetes mellitus and diabetes-related chronic complications

**DOI:** 10.1042/BSR20241307

**Published:** 2025-05-13

**Authors:** Farjana Yasmin, Kim San Tang

**Affiliations:** 1 Center for Pharmacometrics and Systems Pharmacology, Department of Pharmaceutics, College of Pharmacy, University of Florida, Orlando, FL 32827, U.S.A.; 2 School of Pharmacy, Monash University Malaysia, Jalan Lagoon Selatan, 47500 Bandar Sunway, Selangor, Malaysia

**Keywords:** diabetic cardiomyopathy, diabetes complications, diabetes mellitus, diabetic nephropathy, diabetic neuropathy, polydatin

## Abstract

Diabetes mellitus is a complex metabolic disorder associated with severe complications affecting various organs, including the kidneys, nerves, heart, and blood vessels. Managing these complications remains a significant clinical challenge, necessitating the exploration of novel therapeutic approaches. This review focuses on polydatin, a naturally occurring glycoside from *Polygonum cuspidatum*, highlighting its potential as a multitargeted therapeutic agent against diabetic complications. Evidence indicates that polydatin effectively improves insulin sensitivity, lowers blood glucose levels, and exhibits antioxidant properties. In diabetic nephropathy, polydatin has been shown to reduce oxidative stress, inflammation, and podocyte apoptosis, thereby preserving renal function. Furthermore, it enhances mitochondrial function and Sirt1 expression in diabetic neuropathy, promoting nerve regeneration and alleviating pain. In cardiac studies, polydatin protects against diabetic cardiomyopathy by enhancing autophagy and reducing oxidative stress, ultimately improving cardiac function. Additionally, polydatin restores endothelial function in vascular complications associated with diabetes. Polydatin presents a promising natural therapy with the potential to mitigate multiple complications of diabetes through its antioxidant, anti-inflammatory, and cytoprotective effects. Although findings from animal models and *in vitro* studies are promising, further clinical research is essential to validate its efficacy and safety in human subjects. By integrating polydatin into diabetes management strategies, there is potential for improved health outcomes and quality of life for individuals affected by this chronic condition.

## Introduction

Diabetes mellitus is a chronic metabolic condition characterized by hyperglycaemia, resulting from either insufficient insulin production or the body’s inability to respond effectively to insulin [1]. This persistent increase in blood glucose levels can lead to various complications if left unmanaged, including nephropathy, neuropathy, retinopathy, and peripheral vascular disease, which significantly affect a person’s quality of life [2]. The global prevalence of diabetes is rapidly increasing, with an estimated 537 million individuals currently living with the condition [3]. This number is expected to rise to 783 million by 2045, as the incidence of type 2 diabetes, particularly in middle- and low-income countries, continues to grow [3]. The economic burden of diabetes is also escalating, with the global cost of managing diabetes-related illnesses projected to reach a staggering 1,054 billion USD by 2045 [4]. Effective management and prevention strategies are crucial to mitigating these impacts.

The landscape of diabetes management has evolved significantly over the years, with a variety of anti-diabetic medications providing effective blood glucose control. These medications include classes such as metformin, sulfonylureas, SGLT2 inhibitors, and GLP-1 receptor agonists, all of which are generally effective in managing blood sugar [5]. Although conventional medications have been shown to control glucose levels, they are often accompanied by side effects, such as gastrointestinal disturbances, weight gain, and an increased risk of hypoglycaemia, which can negatively affect patient adherence and overall quality of life [6]. As a result, there is growing interest in alternative approaches, such as natural products and herbal supplements, which are often perceived to offer a more gentle approach with fewer side effects [7].

*Polygonum cuspidatum*, commonly known as Japanese knotweed, is a traditional Chinese medicinal herb used to treat various ailments, including cough, fever, hypertension, and pain [8]. This herb contains a natural precursor to resveratrol called 3,4,5-trihydroxystilbene-3-D-glucoside, or polydatin [9]. Research indicates that polydatin can effectively regulate lipid and glucose metabolism and possesses antioxidant properties that help protect the body from oxidative stress and complications associated with diabetes [10]. Although Ke et al. (2023) [8] provide a comprehensive review of the pharmacological properties of *Polygonum cuspidatum*, the potential therapeutic benefits of polydatin in diabetes were only briefly addressed. In this review, we evaluate the scientific literature on the protective effects of polydatin in treating diabetes and its related complications, focusing on both *in vitro* and animal models. This review aims to provide a comprehensive understanding of polydatin’s mechanisms and therapeutic potential. Table 1 summarizes the protective effects of polydatin against various diabetes-related complications.

**Table 1:** Pharmacological properties of polydatin.

References	Affected organ/ abnormalities	Experimental subjects	Diabetes induction	Polydatin doses	Polydatin route of administration	Polydatin duration of treatment	Effects
Yousef et al., 2021 [11]	Pancreatic injury	Wistar albino rats, male, 10 weeks old, 120 ± 10 g	STZ, 50 mg/kg, i.p.	50 mg/kg/day	Oral gavage	4 weeks	↓blood glucose, ↑serum insulin ↓TG, LDL-C, vLDL-C, free fatty acids (serum) ↑HDL-C (serum) ↓LPO, IL-1β (pancreas) ↑CAT, SOD, GPx, GSH (pancreas)
		RINm5F cells	H_2_O_2_, 100 μM (oxidative stress model)	20 and 40 μg/ml	Cell culture	24 h	↑cell viability, ↓ROS, ↑HO-1 ↓apoptosis (↑Bcl-2 mRNA, ↓Bax mRNA, ↑Bcl-2/Bax ratio), ↑Ins1 mRNA
Abd El-Hameed et al., 2021 [12]	Liver injury	Wistar Albino rats, male, 120–140 g	STZ, 50 mg/kg, i.p.	50 mg/kg/day	Oral gavage	4 weeks	↓AST, ALT (serum) ↑GSH, SOD, CAT, GPx, G6PD (liver) ↓MDA (liver) ↑GLUT2 and GCK mRNAs (liver) ↓TNF-α and IL-1β mRNAs (liver) ↓liver histological abnormalities
Hao et al., 2014 [13]	Glucose and lipid metabolism	Sprague Dawley rats, 8 weeks old, 130 ± 15 g	STZ, 30 mg/kg, i.p.	75 and 150 mg/kg, 6 days/week	Oral gavage	8 weeks	↓TC, TG, LDL-C (serum) ↑p-Akt, p-GSK-3β, glycogen (liver) ↓G6Pase, ↑GCK (liver) ↓SREBP-1c, ↑LDLR (liver)
		HepG2 cells	Palmitic acid, 0.25 mM, 12 h (insulin-resistant model)	10, 20, and 40 µM	Cell culture	2 h pre-treatment	↑glucose uptake and consumption ↓lipid accumulation ↑p-Akt, p-IRS-1/2, p-GSK-3β, GCK, ↓G6Pase ↓SREBP-1c, ↑LDLR
Hao et al., 2018 [14]	Glucose and lipid metabolism	HepG2 cells	Insulin, 100 nM, 24 h (insulin-resistant model)	10, 20, and 40 µM	Cell culture	2 h pre-treatment	↑glucose metabolism (↑p-AMPK, p-Akt, p-GSK-3β) ↑lipid metabolism (↓SREBP-1c, ↑p-ACC, ↑LDLR)
Wang et al., 2016 [15]	Glucose and lipid metabolism/liver injury	db/db mice (diabetic) vs. C57BL/6 mice (wild type), female, 6 weeks old	Genetically modified	100 mg/kg, 6 days/week	Oral gavage	4 weeks	↓FBG, ↑glycogen ↓TC, TG, LDL-C ↑LDLR, GCK, ↓PCSK9 ↓liver histological abnormalities
		HepG2 cells	Palmitic acid, 0.25 mM, 24 h (insulin-resistant model)	5, 10, 20, and 40 µM	Cell culture	1 h pre-treatment	↑LDLR, GCK, ↓PCSK9
Abd El-Hameed et al., 2020 [16]	Nephropathy	Wistar albino rats, male, 120–140 g	High-fat diet, 4 weeks; nicotinamide, 110 mg/kg, i.p; STZ, 50 mg/kg, i.p.	50 mg/kg/day	Oral gavage	4 weeks	↓FBG, PBG, HbA1c , AGEs ↑insulin, albumin (serum) ↓uric acid, SCr, BUN (serum) ↓MDA (kidney) ↑SOD, CAT, GSH (kidney) ↓NF-κB, COX-2 (kidney) ↓TNF-α, IL-6, IL-8 (serum) ↑IL-10 (serum)
Huang et al., 2015 [17]	Nephropathy	Sprague-Dawley rats, male, 210 ± 20 g	STZ, 50 mg/kg, i.p.	150 mg/kg/day	Oral gavage	12 weeks	↓FBG, KW/BW ratio, BUN, SCr, 24 h UP ↑Sirt1, Nrf2 (kidney) ↓RAGE (kidney) ↑SOD, ↓MDA (kidney)
		GMCs	AGEs, 100 μg/mL	5, 10, and 20 μM	Cell culture	2 h pre-treatment	↓Keap1, ROS, FN, TGF-β1 ↑Sirt1, Nrf2, ARE-binding activity of Nrf2 ↑HO-1, SOD1
Niu et al., 2019 [18]	Nephropathy	Sprague-Dawley rats, 225–250 g	STZ, 65 μM, i.p.	20, 40, and 80 mg/kg/day		8 weeks	↓FBG, BW, KW/BW ratio, 24 h UP, SCr, BUN, interstitial injury score, CTGF, FN, collagen I ↓IL-1β, IL-6, MCP-1 (serum, kidney) ↓p-IkB-α/IkB-α ratio, p-p65/p65 ratio, TLR4
		NRK-52E cells	HG, 0.3 mM	0.1, 0.2, and 0.4 mM	Cell culture		↓apoptosis, CTGF, FN, collagen I, cleaved caspase-3 ↑Bcl-2 ↓IL-1β, IL-6, MCP-1 ↓p-IkB-α/IkB-α ratio, p-p65/p65 ratio, TLR4
Xie et al., 2012 [19]	Nephropathy	Sprague–Dawley rats, male, 200 ± 10 g	STZ, 60 mg/kg, i.v.	150 mg/kg/day	i.g.	12 weeks	↓KW, KW/BW ratio ↓BUN, SCr, 24 h UP ↓FN (kidney) ↑IkB-α ↓ICAM-1, TGF-β (kidney)
		GMCs	HG, 30 mM, up to 24 h	10, 20, and 40 μM	Cell culture	Up to 12 h pre-incubation	↓FN, NF-κB p65 ↑IkB-α ↓ICAM-1, TGF-β
Chen et al., 2016 [20]	Nephropathy	BKS db/db mice (diabetic) vs. C57 mice (nondiabetic control), female	Genetically modified	100 mg/kg, 6 days/week	Oral gavage	4 weeks	↓FBG, KW/BW ratio, BUN, SCr, 24 h UP ↓renal damage, FN, ICAM-1, SphK1, S1P (kidney)
		GMCs	AGEs, 150 μg/ml, up to 24 h	10, 20, and 40 μM	Cell culture		↓FN, ICAM-1, SphK1, S1P, p-c-Jun, p-c-Fos, transcriptional activity of AP-1
Chen et al., 2020 [21]	Nephropathy/ renal fibrosis	C57BL/6 mice, male, 6–8 weeks old, 20 ± 2 g, specific pathogen-free grade	STZ, 50 mg/kg, i.p., 5 consecutive days	100 mg/kg, 6 days per week	Oral gavage	12 weeks	↓FBG, KW/BW ratio, SCr, BUN, 24 h UP, weight loss, glomerular hypertrophy, mesangial expansion, deposition of collagen fibers in glomeruli, Nox4, FN, ICAM-1, PAI-1, CTGF, collagen IV, TGF-β, MDA ↑Cx32, SOD
		GMCs	HG, 30 mM, 24 h	10, 20, and 40 μM	Cell culture	2 h pre-treatment	↓FN, ICAM-1, superoxide, H_2_O_2_, mitochondrial superoxide, Nox4 ↑Cx32, K48-linked polyubiquitination
Gong et al., 2017 [22]	Nephropathy	C57BL/6 J mice, male, 6 weeks old	STZ, 50 mg/kg, i.p., 5 consecutive days	100 and 200 mg/kg, 6 days/week	Oral gavage	8 weeks	↓FBG, BUN, SCr, 24 h UP, GSP ↑Nrf2, CKIP-1, HO-1, SOD1 (kidney) ↓Keap1, MDA, FN, ICAM-1 (kidney) ↓MDA, ↑total SOD activity (serum, kidney) ↓lipid hydroperoxides (serum, urine)
		GMCs	HG, 30 mM, 6 h	10, 20 and 40 μM	Cell culture	2 h pre-treatment	↓Keap1, FN, ICAM-1, superoxide, H_2_O_2_ ↑Nrf2, ARE-binding activity of Nrf2, CKIP-1, ↑HO-1, SOD1
Ni et al., 2017 [23]	Nephropathy	KKAy mice (diabetic) and C57BL/6 J mice (nondiabetic control), male, 9–11 weeks old	Genetically modified, high-fat diet	100 mg/kg/day	Oral	8 weeks	↓FBG, KW/BW ratio, 24 h UP, UAE, SCr, BUN ↓podocyte apoptosis, mesangial expansion ↑nephrin, podocin
		MPC5 cells	HG, 30 mM	25 mM	Cell culture	24, 48, and 72 h co-treatment	↓apoptosis (↓ cytochrome C, cleaved caspase-3 and -9) ↓Drp1, ROS
Bheereddy et al., 2020 [24]	Neuropathy	Sprague–Dawley rats, male, 250–300 g	STZ, 55 mg/kg, i.p.	25 and 50 mg/kg	Oral gavage	Last 2 weeks of an 8-week study	↓nerve functional defects ↑SIRT1, PGC-1α, Nrf2, TFAM
		Neuro2a cells	HG, 30 mM, 24 h	5, 10, and 20 µM		24 h co-treatment	↓ROS, mitochondria dysfunction ↑Nrf2, NQO1, SOD2 ↑SIRT1, PGC-1α, Nrf2, TFAM ↑neuritogenesis
Chen et al., 2021 [25]	Neuropathy	Sprague Dawley rats, male, 180 ± 20 g	STZ, 70 mg/kg, i.p.	25 and 50 mg/kg/day	i.p.	Up to 4 weeks	↑morphological repair of crushed sciatic nerves
		RSC96 cells	MGO, 1.65 mM, 6 h (short term) and 24 h (long term)	50, 100, and 150 µM	Cell culture	12 h pre-treatment	↑cell viability, MMP ↓LDH release, ROS, apoptosis ↑Nrf2, ↓Keap1 ↑GLO1, ↓RAGE
Tan et al., 2020 [26]	Cardiomyopathy	Sprague–Dawley rats, male, 200–220 g	High-fat diet for 4 weeks followed by STZ, 30 mg/kg, i.p. for 2 times in 24-h interval	100 mg/kg/day	Oral gavage	8 weeks	↓FBG, TC, TG, glycated hemoglobin ↑BW, fasting insulin ↓cardiac dysfunction, cardiac hypertrophy, interstitial fibrosis ↓myocardial oxidative stress (↓MDA, 4-HNE, ROS, Nox2, Nox4) ↓myocardial NF-κB, p65, IL-1β, IL-6, TNF-α, VCAM-1
		H9c2 cells	HG, 30 mM, 24 h	40 µM	Cell culture	24 h co-treatment	↓ROS, Nox2, Nox4 ↓ NF-κB, IkB-α, p65, IL-1β, IL-6, TNF-α, VCAM-1
Yu et al., 2018 [27]	Cardiomyopathy	Sprague–Dawley rats, male, 220–250 g	STZ, 50 mg/kg, i.p. for 3 consecutive days	20 mg/kg/day	Oral gavage	3 consecutive days and once again prior to the MI/R operation	↑cardiac function, ↓myocardial infarct size ↓apoptosis, caspase-3, plasma CK and LDH ↓myocardial oxidative/nitrative stress (↓superoxide, MDA, gp91phox, iNOS, NO, nitrotyrosine; ↑p-eNOS) ↑Notch/Hes1-Pten/Akt signaling
Wu et al., 2015 [28]	Vascular complications	Thoracic aortas rings from Sprague–Dawley rats, male and female, 250–260 g	HG, 55 mM, 6 h	1, 3, and 10 μM	Organ bath	6 h co-treatment	↑endothelium-dependent relaxation ↓histological damage of endothelial cells ↑PPARβ, eNOS, NO ↓iNOS
Shah et al., 2023 [29]	Vascular complications	Aortas from Sprague–Dawley rats, male, 13 weeks old	STZ, 60 mg/kg, i.p. for 7 days	10 μM	Organ bath	6 h co-treatment	↓acetylcholine-induced vasodilation of isolated aortas
		Thoracic aortas rings from Sprague–Dawley rats, male, 13 weeks old	HG, 30 mM, 6 h	10 μM	Organ bath	6 h co-treatment	↓histological damage ↑eNOS, ↓iNOS ↓ROS ↓NLRP3, VCAM-1, caspase-1, IL-1β
		HUVECs	HG, 30 mM, 48 h	10 μM	Cell culture	48 h co-treatment	↑cell viability, MMP ↓pyroptosis ↓NLRP3, VCAM-1, caspase-1, IL-1β ↓Drp1, ↑p-Drp1/Drp-1 ratio
Pang et al., 2017 [30]	Vascular complications	HUVECs	MGO, 200 μM, 1 h or 24 h	25, 50, and 100 μM	Cell culture	2 h pre-treatment	↓apoptosis, caspase-3, Bax/Bcl-2 ratio, ROS ↑SOD, CAT, GPx, MMP, p-Akt

4-HNE, 4-hydroynonenal; ACC, acetyl-CoA carboylase; AGEs, advanced glycation end products; ALT, alanine transaminase; AMPK, AMP-activated protein kinase; AP-1, activator protein 1; ARE, antioidant response element; AST, aspartate transaminase; Ba, Bcl-2-associated X protein; BUN, blood urea nitrogen; BW, body weight; CAT, catalase; CKIP-1, casein kinase 2 interacting protein 1; COX-2, cyclooygenase-2; CTGF, connective tissue growth factor; C32, connein 32; Drp1, dynamin-related protein 1; eNOS, endothelial nitric oide synthase; FBG, fasting blood glucose; FN, fibronectin; G6Pase, glucose 6-phosphatase; G6PD, glucose-6-phosphate dehydrogenase; GCK, glucokinase; GLO1, glyoalase 1; GLUT2, glucose transporter 2; GMCs, glomerular mesangial cells; GP, glutathione peroidase; GSH, glutathione; GSK-3β, glycogen synthase kinase-3 beta; H2O2, hydrogen peroide; HbA1c, haemoglobin A1C; HDL-C, high-density lipoprotein cholesterol; HES1, hairy and enhancer of split 1; HG, high glucose; HO-1, heme oygenase 1; ICAM-1, intercellular adhesion molecule 1; IL, interleukin; iNOS, inducible nitric oide synthase; Ins1, insulin 1; i;p, intraperitoneal; IRS, insulin receptor substrate; Keap1, Kelch-like ECH associated-protein 1; KW, kidney weight; LDH, lactate dehydrogenase; LDL-C, low-density lipoprotein cholestero; LDLR, low-density lipoprotein receptor; LPO, lipid peroidation; MCP-1, monocyte chemoattractant protein-1; MDA, malondialdehyde; MGO, methylglyoal; MI/R, myocardial ischemia and reperfusion; MMP, mitochondria membrane potential; NF-κB, nuclear factor kappa B; NLRP3, NLR family pyrin domain containing 3; NO, nitric oide; No, NADPH oidase; NQO1, NAD(P)H dehydrogenase [quinone] 1; Nrf2, nuclear factor E2-related factor 2; PAI-1, plasminogen activator inhibitor-1; PBG, postprandial blood glucose; PCSK9, proprotein convertase subtilisin/Kein type 9; PGC-1α, peroisome proliferator-activated receptor gamma coactivator-1 alpha; PPARβ, peroisome proliferator-activated receptor beta; Pten, phosphatase and tensin homolog; RAGE, receptor for advanced glycation end products; ROS, reactive oygen species; S1P, sphingosine 1-phosphate; SCr, serum creatinine; Sirt1, sirtuin 1; SOD, superoide dismutase; SphK1, sphingosine kinase 1; SREBP-1c, sterol regulatory element-binding protein 1c; STZ, streptozotocin; TC, total cholesterol; TFAM, transcription factor A, mitochondrial; TG, triglycerides; TGF-β1, transforming growth factor beta 1; TLR4, toll-like receptor 4; TNF-α, tumour necrosis factor alpha; UAE, urinary albumin ecretion; 24-h UP, 24-h urine protein; VCAM-1, vascular cell adhesion molecule 1; vLDL-C , very low-density lipoprotein cholesterol.

## Polydatin and insulin sensitivity

Insulin resistance and decreased insulin production are the primary pathogenic mechanisms underlying type 2 diabetes, significantly disrupting glucose metabolism and triggering a cascade of oxidative stress and inflammatory reactions that exacerbate the condition [31]. This resistance to insulin not only impairs glucose uptake but also contributes to dyslipidaemia, endothelial dysfunction, and the subsequent development of atherosclerotic plaques, which heightens cardiovascular risk [32]. Recent studies have highlighted the potential benefits of polydatin in this context, showing its ability to improve insulin sensitivity and promote pancreatic insulin production [11,16,33]. By enhancing insulin signalling pathways and reducing inflammatory markers, polydatin may help restore normal glucose metabolism and mitigate some of the harmful effects associated with insulin resistance [12–14].

After administering polydatin to diabetic rats, significant improvements were observed in various metabolic parameters, indicating its potential efficacy in managing diabetes [11,13,16–19,26]. Specifically, measurements such as the homeostatic model assessment for insulin resistance, haemoglobin A1C, and fasting blood glucose levels showed notable decreases, while insulin levels experienced a marked increase. These findings suggest that polydatin may exert its anti-diabetic effects primarily by enhancing insulin sensitivity and stimulating pancreatic insulin production. By improving insulin action in peripheral tissues and promoting the pancreas’s ability to secrete insulin, polydatin appears to address key underlying issues associated with insulin resistance.

In type 2 diabetes, abnormalities in lipid and glucose metabolism are frequently observed due to reduced insulin sensitivity, which exacerbates metabolic dysregulation and contributes to the progression of the disease [31]. Studies have demonstrated that polydatin can enhance glucose metabolism and uptake while simultaneously decreasing lipid accumulation in insulin-resistant human hepatoma HepG2 cells, indicating its potential as a therapeutic agent in managing metabolic dysfunctions associated with diabetes [13,14]. The beneficial effects of polydatin appear to be mediated through key signalling pathways involving proteins such as Akt and AMP-activated protein kinase (AMPK). Akt activation plays a critical role in promoting glucose uptake by facilitating the translocation of glucose transporter proteins to the cell membrane, while AMPK activation enhances cellular energy balance and inhibits lipid synthesis [34,35]. By modulating these pathways, polydatin not only improves glucose utilization but also mitigates lipid accumulation, thereby addressing two central aspects of metabolic dysfunction in type 2 diabetes.

Activation of Akt plays a crucial role in promoting glycogen synthesis by enhancing the activity of glucokinase (GCK), inhibiting glucose-6-phosphatase, and suppressing glycogen synthase kinase 3 beta (GSK-3β) [13,15,36]. However, it is important to note that impaired Akt signalling is commonly observed in diabetic tissues, resulting in compromised glucose metabolism and reduced glycogen storage [37]. Research studies using insulin-resistant cell models and diabetic animals have shown that polydatin can effectively improve glucose metabolism by elevating the phosphorylation levels of key proteins involved in these processes, including AMPK, Akt, GSK-3β, and insulin receptor substrate 1 or 2 (IRS1/2) [13–15,33]. By enhancing the phosphorylation of these proteins, polydatin facilitates hepatic glycogen synthesis, thereby helping to control blood glucose levels more effectively.

In the liver of diabetic rats, the expression of GCK is notably lower, while glucose-6-phosphatase levels are elevated compared with normal controls, contributing to dysregulated glucose metabolism [13]. Research has demonstrated that polydatin treatment can wholly or partially reverse the expression levels of these critical proteins, with effects observed at various dosages [13]. In another study, administering polydatin at a dosage of 50 mg/kg body weight has been shown to significantly affect carbohydrate metabolism by reducing fasting blood glucose levels while markedly increasing the activities of carbohydrate metabolism enzymes, including pyruvate kinase and succinate dehydrogenase, when compared with diabetic rats [12]. These findings indicate that polydatin not only enhances insulin sensitivity and production but also plays a role in restoring the balance of key enzymes involved in glucose metabolism.

Dyslipidaemia is a prevalent condition among patients with type 2 diabetes, typically characterized by elevated levels of triglycerides and low-density lipoprotein cholesterol (LDL-C), along with decreased levels of high-density lipoprotein cholesterol (HDL-C) [38]. This lipid imbalance can exacerbate the risk of cardiovascular complications associated with diabetes. Studies have shown that polydatin has a beneficial impact on lipid profiles in diabetic rats, effectively reducing free fatty acids, triglycerides, LDL-C, very low-density lipoprotein cholesterol, and total cholesterol levels [11,13,15,26]. Concurrently, polydatin treatment has been associated with a significant increase in HDL-C levels, which is particularly important given HDL-C’s protective role against atherosclerosis and cardiovascular disease [11,13]. These findings suggest that polydatin may help address the lipid abnormalities associated with diabetes, presenting a potential therapeutic strategy for managing dyslipidemia in this patient population.

Polydatin enhances lipid metabolism through several mechanisms that target key regulatory proteins involved in lipid homeostasis. One of the primary actions of polydatin is the phosphorylation of acetyl-CoA carboxylase, a key enzyme involved in fatty acid synthesis and oxidation, as demonstrated in insulin-resistant HepG2 cells [14]. This phosphorylation promotes fatty acid oxidation, thereby reducing lipid accumulation. Additionally, polydatin effectively decreases the nuclear levels of sterol regulatory element-binding protein 1 c (SREBP-1c), a transcription factor that stimulates cholesterol synthesis [14]. By lowering SREBP-1c levels, polydatin helps to inhibit excessive cholesterol production in the liver. Furthermore, polydatin has been shown to enhance the expression of low-density lipoprotein receptor (LDLR), which are crucial for the clearance of LDL-C from the bloodstream [14]. Increased LDLR levels facilitate the excretion of LDL-C, thereby contributing to improved lipid profiles. Moreover, proprotein convertase subtilisin/kexin type 9 (PCSK9), which is primarily synthesized in the liver, plays a critical role in the degradation of LDLR [15].

Polydatin exerts a beneficial impact on glucose and lipid metabolism by down-regulating PCSK9 while simultaneously up-regulating GCK and LDLR in insulin-resistant HepG2 cells [15]. In a study involving db/db mice, treatment with polydatin at a dosage of 100 mg/kg resulted in significantly increased levels of LDLR and GCK, coupled with a notable inhibition of PCSK9 expression [15]. These findings highlight polydatin’s potential to enhance lipid clearance and glucose metabolism in diabetic conditions. Furthermore, molecular docking studies have indicated that polydatin can bind to the active pocket of the PCSK9 crystal structure (PDB code: 2p4e), forming stable hydrogen bonds that modify the conformation of PCSK9 [15]. This interaction is crucial as it limits PCSK9’s ability to bind to LDLR, thereby reducing their degradation and promoting increased availability of LDLR on the hepatocyte surface.

**Figure 1:**
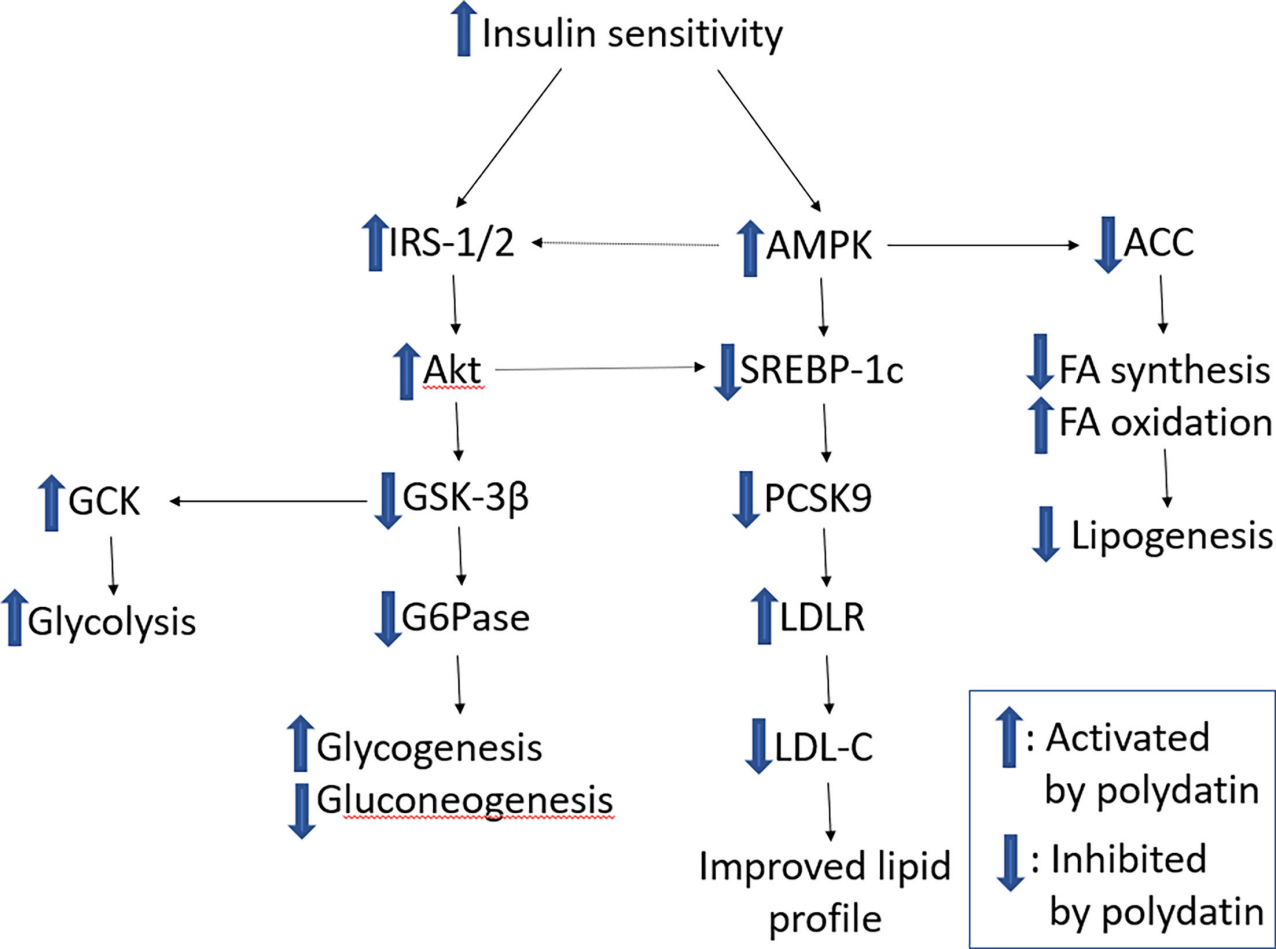
The effects of polydatin on glucose and lipid metabolism. Abbreviations: ACC, acetyl-CoA carboxylase; AMPK, AMP-activated protein kinase; FA, fatty acid; G6Pase, glucose 6-phosphatase; GCK, glucokinase; GSK-3β, glycogen synthase kinase-3 beta; IRS-1/2, insulin receptor substrate-1 or 2; LDL-C, low-density lipoprotein cholesterol; LDLR, low- density lipoprotein receptor; PCSK9, proprotein convertase subtilisin/kexin type 9; SREBP-1c, sterol regulatory element-binding protein 1 c.

The above findings suggest that polydatin improves insulin sensitivity through various mechanisms, including the enhancement of glucose metabolism and lipid profiles (Figure 1). Its ability to down-regulate harmful proteins like PCSK9 and up-regulate beneficial ones such as GCK and LDLR indicates a multifaceted approach to metabolic regulation [13–15]. Moreover, the modulation of key signalling pathways, including those involving Akt and AMPK, underscores its potential role in addressing insulin resistance [14]. Despite these promising findings, further exploration into the specific molecular pathways influenced by polydatin is needed to fully understand its role in insulin production and its therapeutic potential for type 2 diabetes management.

## Protective effects of polydatin in diabetic hepatopathy

Diabetic hepatopathy is a significant complication of diabetes, often characterized by liver damage and dysfunction that can lead to serious health issues, including non-alcoholic fatty liver disease and hepatic fibrosis [39]. This condition is marked by a range of pathological changes in liver tissue, such as steatosis, inflammation, and fibrosis, which compromise liver function and exacerbate metabolic dysregulation. These alterations not only impair the liver’s ability to process nutrients and detoxify harmful substances but also contribute to systemic metabolic complications.

Polydatin therapy has shown promise in improving liver tissue integrity and protecting against diabetes-induced histological abnormalities. The assessment of liver function often relies on biochemical markers, particularly the activities of aspartate transaminase (AST) and alanine transaminase (ALT), with elevated levels of these enzymes suggesting liver damage. Polydatin treatment has been shown to significantly reduce ALT and AST activities in diabetic rats, suggesting a protective effect on liver function [12]. Additionally, polydatin therapy resulted in significantly increased serum concentrations of albumin and total protein, further indicating its positive influence on liver health [12].

In hyperglycaemic states, the function of antioxidant enzymes is often significantly reduced, particularly that of catalase (CAT) and superoxide dismutase (SOD). This reduction leads to an accumulation of reactive oxygen species (ROS), which can inflict oxidative damage on liver tissues, contributing to the progression of diabetic complications [40]. However, polydatin has demonstrated a remarkable capacity to combat oxidative stress, effectively elevating the levels of glutathione (GSH) and enhancing the activities of several key antioxidant enzymes, including SOD, glutathione peroxidase (GPx), CAT, and glucose-6-phosphate dehydrogenase (G6PD) [12]. This boost in antioxidant activity plays a crucial role in mitigating oxidative damage, as it helps to neutralize excess ROS, thereby preserving cellular integrity and function. Moreover, the enhancement of antioxidant defence mechanisms has been associated with a significant decrease in malondialdehyde (MDA) production, a well-established marker of lipid peroxidation that indicates oxidative stress within cells [12].

In addition to its antioxidant properties, polydatin exhibited notable anti-inflammatory effects by suppressing the mRNA levels of inflammatory cytokines such as tumour necrosis factor-alpha (TNF-α) and interleukin-1 beta (IL-1β) in the liver tissues of diabetic rats [12]. TNF-α and IL-1β are key mediators of the inflammatory response and are often elevated in diabetic conditions, contributing to the pathogenesis of insulin resistance and liver dysfunction [41]. By reducing the expression of these pro-inflammatory cytokines, polydatin may help mitigate the chronic inflammation that exacerbates liver injury and impairs metabolic function.

Furthermore, polydatin was found to increase the expression levels of glucose transporter-2 (GLUT2) and GCK genes in diabetic animals [12]. GLUT2 plays a crucial role in glucose homeostasis by facilitating the transport of glucose into cells, particularly in the liver and pancreatic beta cells [39]. An up-regulation of GLUT2 can lead to improved glucose uptake, which is essential for maintaining normal blood sugar levels. Similarly, GCK is a key enzyme in glucose metabolism that catalyses the phosphorylation of glucose to glucose-6-phosphate, a vital step in glycolysis and glycogen synthesis [42]. Thus, increased expression of GCK not only enhances the capacity of cells to utilize glucose but also aids in lowering blood glucose levels, thus contributing to better glycaemic control.

In short, polydatin exhibits protective effects against diabetic hepatopathy by improving liver function, reducing oxidative stress, and suppressing inflammation. These multifaceted actions contribute to the overall health of the liver, which is often compromised in individuals with diabetes due to the accumulation of fat, inflammation, and oxidative damage. By enhancing liver function, polydatin not only aids in the metabolic processing of glucose but also supports the detoxification processes essential for maintaining homeostasis.

## Protective role of polydatin in mitigating pancreatic damage

Oxidative stress is a significant contributor to the malfunction and damage of pancreatic β-cells, playing a crucial role in the pathophysiology of type 2 diabetes [43]. In healthy individuals, pancreatic β-cells are responsible for producing and releasing insulin, a hormone essential for regulating blood glucose levels. However, in the presence of chronic hyperglycaemia, often resulting from obesity and sedentary lifestyles, these cells face increased oxidative stress due to elevated levels of ROS [44]. This oxidative burden impairs the β-cells’ ability to function optimally, leading to cellular dysfunction and eventual apoptosis [45]. Consequently, as these cells become damaged and lose their functional capacity, they are unable to produce and release sufficient insulin to counteract peripheral insulin resistance, which is a hallmark of type 2 diabetes [46].

Given this context, Yousef et al. [11] investigated the protective benefits of polydatin against oxidative damage in pancreatic β-cells using both *in vivo* and *in vitro* models [11]. Their comprehensive study demonstrated that treatment with polydatin led to a significant increase in the levels of crucial antioxidant enzymes, including CAT, GPx, and SOD in diabetic rats. This enhancement in antioxidant enzyme activity strongly suggests that polydatin may effectively mitigate oxidative stress within the pancreatic environment, which is vital for preserving the functionality of β-cells. Additionally, in experiments involving insulin-producing RINm5F cells subjected to hydrogen peroxide treatment, polydatin was found to improve cell viability and significantly reduce the accumulation of ROS, highlighting its protective role in cellular contexts prone to oxidative stress [11].

Moreover, pro-inflammatory cytokines such as IL-1β and TNF-α play a critical role in the progression of diabetes by inhibiting glucose-induced insulin production and impairing β-cell functionality, thereby contributing to the worsening of hyperglycaemia and the overall metabolic dysfunction characteristic of the disease [41]. These cytokines create a detrimental inflammatory environment within the pancreas, leading to β-cell apoptosis and reduced insulin secretion, which further exacerbates insulin resistance. In this context, the research by Yousef et al. [11] revealed that polydatin has a significant capacity to modulate inflammatory responses in diabetic rats by effectively preventing the elevation of the pro-inflammatory cytokine IL-1β [11]. This action not only highlights polydatin’s anti-inflammatory properties but also suggests its potential to preserve β-cell function by mitigating the inflammatory damage that can lead to reduced insulin production.

These findings collectively suggest that polydatin may play a protective role in maintaining β-cell function and preventing oxidative damage, primarily through its potent antioxidant and anti-inflammatory properties. By enhancing the activity of key antioxidant enzymes and reducing levels of harmful pro-inflammatory cytokines, polydatin appears to create a more favourable environment for β-cells, potentially enhancing their insulin-producing capacity and safeguarding them from the detrimental effects of oxidative stress. However, it is essential to note that the study did not elucidate the specific underlying mechanisms responsible for β-cell protection beyond these effects, leaving questions about the precise pathways through which polydatin exerts its benefits [11]. Understanding these mechanisms is critical for maximizing its therapeutic potential and developing targeted interventions.

## Protective potential of polydatin in diabetic nephropathy

Diabetic nephropathy is a severe microvascular complication of diabetes mellitus and has become the leading cause of kidney failure, significantly impacting patient morbidity and mortality [47]. This condition is characterized by progressive kidney damage resulting from a combination of factors, including hyperglycaemia, oxidative stress, and inflammation, which ultimately lead to a decline in renal function. Evidence suggests that polydatin can significantly improve renal health by lowering fasting blood glucose levels, which is crucial for mitigating the adverse factors contributing to kidney damage in diabetic conditions [16–18,20–23].

In various diabetic animal models, treatment with polydatin has been associated with reductions in key metrics indicative of renal impairment, such as the kidney weight/body weight ratio, urine protein, urinary albumin excretion, serum creatinine, and blood urea nitrogen levels [17–19,23]. These improvements highlight polydatin’s potential as a therapeutic agent in managing diabetic nephropathy. Furthermore, polydatin treatment has been shown to reduce interstitial damage scores, which reflect the extent of tissue injury in the kidneys, and down-regulate the levels of crucial fibrotic markers such as connective tissue growth factor, fibronectin, and collagen I in streptozotocin (STZ)-induced diabetic rats [18]. These markers are associated with renal fibrosis, and their reduction suggests that polydatin may help prevent or reverse the fibrotic processes that characterize diabetic nephropathy.

Additionally, polydatin therapy resulted in the restoration of podocin and nephrin protein levels, which are vital for maintaining the structure and function of podocytes, the specialized cells that line the kidney’s filtration barrier [23]. This restoration normalizes the morphology of foot processes and podocyte slit pores in the kidneys of KKAy mice, indicating that polydatin may help preserve the integrity of the glomerular filtration barrier, thereby enhancing renal function and preventing the progression of nephropathy. Collectively, these findings underscore the potential of polydatin not only to improve metabolic parameters but also to directly protect and restore kidney function in the context of diabetes, warranting further investigation into its mechanisms and clinical applications for diabetic nephropathy.

The apoptosis of podocytes plays a crucial role in the progression of diabetic nephropathy, significantly impacting kidney function and contributing to the overall decline in renal health [48]. One of the underlying mechanisms driving this process is the overproduction of ROS, which occurs as a result of mitochondrial fission. This fission leads to mitochondrial dysfunction, including damage to mitochondrial DNA and impairments in the oxidative respiratory chain, further exacerbating oxidative stress within podocytes [49]. Importantly, research has shown that polydatin has the capacity to reduce apoptosis in high glucose-treated murine podocytes, specifically in MPC5 cells [23]. In a study involving KKAy mice, treatment with polydatin was associated with a notable decrease in the presence of swollen, deformed vesicular mitochondria, indicating improved mitochondrial morphology, as the mitochondria remained elongated and filamentous [23]. This protective effect of polydatin is closely linked to the down-regulation of dynamin-related protein 1 (Drp1) expression. Drp1 is a key regulator of mitochondrial fission; by diminishing its expression, polydatin effectively reduces mitochondrial fission and, consequently, ROS production [50,51]. This reduction in ROS not only alleviates oxidative stress but also leads to decreased rates of cell apoptosis, helping to preserve podocyte viability and function.

In addition to its protective effects on podocytes, polydatin has been shown to activate the nuclear factor erythroid 2-related factor 2 (Nrf2)-ARE anti-oxidative pathway in rat glomerular mesangial cells (GMCs) exposed to advanced glycation end products (AGEs) [17]. AGEs, which are produced in response to hyperglycaemia, are known contributors to the pathogenesis of various diabetic complications, including nephropathy, by promoting oxidative stress and inflammation [52]. Notably, polydatin demonstrated a dose-dependent ability to decrease the expression of kelch-like ECH-associated protein 1 (Keap1), a negative regulator of Nrf2, while simultaneously slightly up-regulating Nrf2 levels in GMCs treated with AGEs [17]. This modulation enhances the localization of Nrf2 in the nucleus, where it can initiate the transcription of various antioxidant genes, thereby increasing the expression of crucial antioxidant enzymes like heme oxygenase-1 (HO-1) and superoxide dismutase 1 (SOD1). This cascade of events helps to reduce ROS overproduction, thereby protecting renal cells from oxidative damage. Polydatin also enhanced the expression of Nrf2 and casein kinase 2 interacting protein-1 (CKIP-1) while reducing Keap1 levels in GMCs subjected to high glucose treatment [22]. This effect boosts the binding activity of Nrf2 to its corresponding ARE element, thereby promoting its transcriptional activity and effectively mitigating oxidative stress-induced damage in diabetic conditions.

Moreover, polydatin treatment significantly mitigated the AGEs-induced overexpression of transforming growth factor-beta 1 (TGF-β1) and fibronectin, both of which are pivotal in the fibrotic processes associated with diabetic nephropathy [17]. This protective effect is attributed to the activation of Sirt1, a protein that not only enhances the Nrf2-ARE pathway but also plays a role in cellular stress responses. Furthermore, polydatin has been shown to dramatically reduce the expression levels of intercellular adhesion molecule-1 (ICAM-1) and fibronectin in GMCs exposed to AGEs [20]. This reduction is attributed to the attenuation of the sphingosine kinase 1-sphingosine-1-phosphate (SphK1-S1P) signalling pathway, as polydatin decreased SphK1 levels and inhibited SphK activity, leading to diminished pro-inflammatory markers. Additionally, polydatin treatment ameliorated kidney damage in db/db mice and suppressed the AGEs-induced phosphorylation of c-Jun and c-Fos, which are critical for the activation of the activator protein-1 (AP-1) transcription factor, resulting in a further decrease in ICAM-1 and fibronectin levels [20]. In STZ-induced diabetic rats, polydatin’s capacity to reduce inflammatory responses by inhibiting the nuclear translocation of nuclear factor kappa B (NF-κB) further underscores its nephroprotective properties [19].

**Figure 2:**
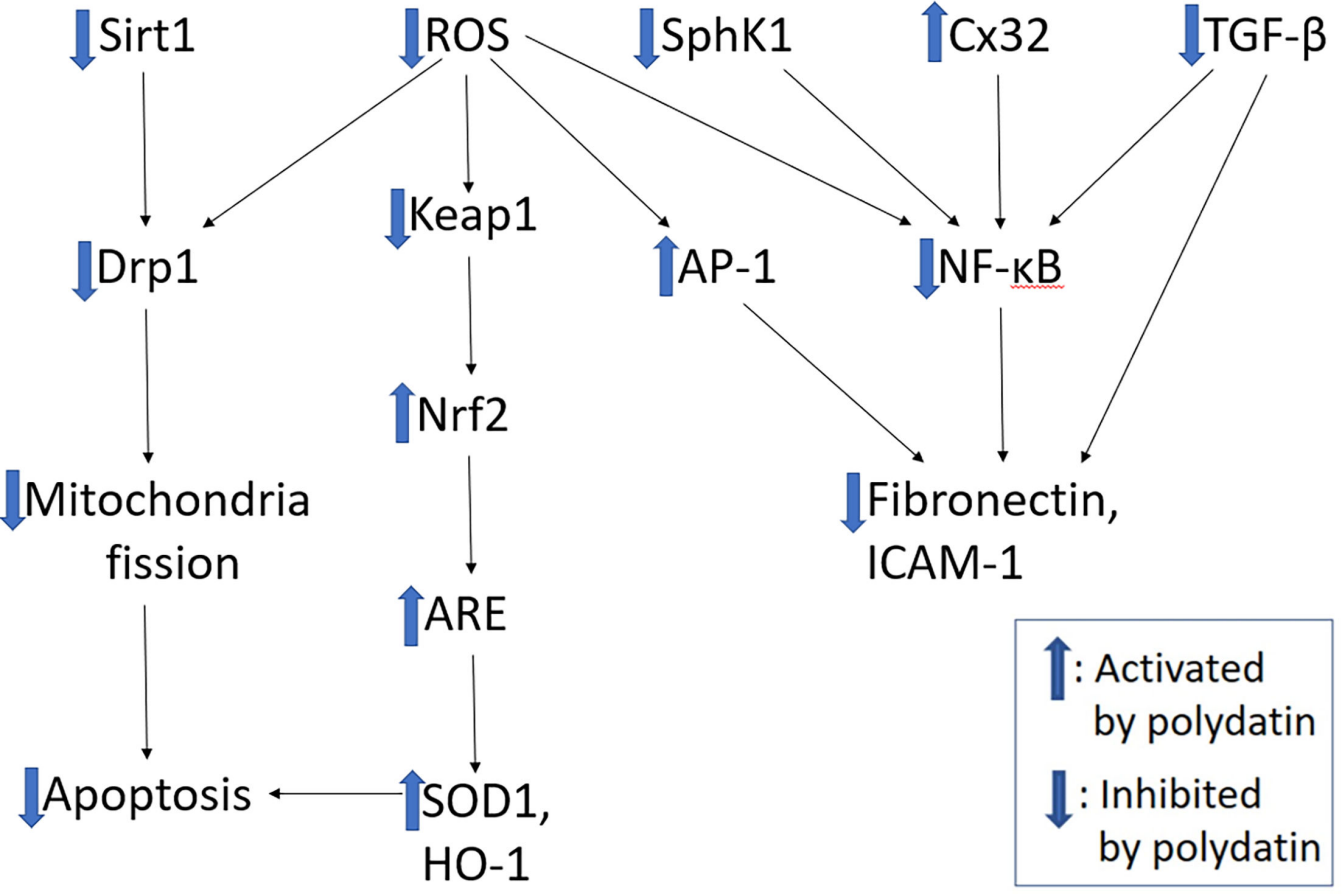
Nephroprotective actions of polydatin. Abbreviations: AP-1, activator protein-1; ARE, antioxidant response element; Cx32, connexin 32; HO-1, heme oxygenase-1; ICAM-1, intercellular adhesion molecule-1; Keap1, Kelch-like ECH associated-protein 1; NF-κB, nuclear factor kappa B; Nrf2, nuclear factor E2-related factor 2; ROS, reactive oxygen species; Sirt1, sirtuin 1; SOD1, superoxide dismutase 1; SphK1, sphingosine kinase 1; TGF-β, transforming growth factor beta.

Further supporting its nephroprotective role, polydatin has been shown to significantly decrease NADPH oxidase (Nox) 4 protein levels in GMCs treated with high glucose by promoting K48-linked polyubiquitination of Nox4, effectively inhibiting ROS production and suppressing the expression of inflammatory markers such as ICAM-1 and fibronectin [21]. In experiments with STZ-induced diabetic mice, administration of polydatin led to improvements in kidney function by enhancing the expression of connexin 32 (Cx32), while simultaneously reducing oxidative stress markers [21]. The restoration of Cx32 is vital, as it facilitates the degradation of Nox4, further lowering oxidative stress levels and slowing the progression of diabetic kidney fibrosis. The nephroprotective effects of polydatin are illustrated in Figure 2.

## Neuroprotective effects of polydatin

Diabetic neuropathy is a complex and debilitating complication of diabetes, marked by reduced expression of Sirt1, a protein that plays a crucial role in cellular stress responses and metabolic regulation [53]. This decrease in Sirt1 levels has been linked to the development of neuropathic pain, a significant and distressing symptom for many individuals with diabetes. Sirt1 is well known for its ability to activate Nrf2, a key transcription factor that helps lower ROS levels by promoting the production of antioxidant enzymes, thereby mitigating oxidative stress within neuronal tissues [54]. The activation of the Nrf2 pathway is essential for dampening both apoptotic and inflammatory processes, which are major contributors to the progression of diabetic neuropathy [55]. Furthermore, hyperglycaemia has been shown to induce mitochondrial dysfunction, a process exacerbated by the modulation of the Nrf2-ARE-Keap1 pathway [56]. Under conditions of elevated glucose, the balance of ROS production and elimination becomes disrupted, leading to oxidative damage that not only impairs mitochondrial function but also exacerbates the symptoms of neuropathy.

To investigate the effects of polydatin on neural function, mouse neuroblastoma Neuro2a cells were exposed to polydatin in a high-glucose environment, a condition that simulates the stress experienced by neurons in diabetic states [24]. The study administered polydatin at doses of 5, 10, and 20 µM, revealing that concentrations of 10 and 20 µM were particularly effective in restoring mitochondrial membrane potential (MMP) and enhancing ATP production, indicating a significant recovery in mitochondrial function. This restoration was further corroborated by findings that polydatin treatment effectively reversed high glucose-induced mitochondrial dysfunction, primarily through the up-regulation of Sirt1 expression, a key regulator of cellular health and longevity. Immunoblotting assays demonstrated that polydatin significantly increased the levels of Nrf2, a critical transcription factor in antioxidant defence, along with its downstream effector proteins, including NAD(P)H quinone dehydrogenase 1 (NQO1) and superoxide dismutase 2 (SOD2), under hyperglycaemic conditions [24,25]. Additionally, exposure to polydatin at a concentration of 20 µM elevated the mRNA levels of mitochondrial transcription factor A (TFAM) and Nrf2, further reinforcing the protective mechanisms activated in response to glucose stimulus [20].

In a study focused on Schwann cell damage induced by methylglyoxal, treatment with polydatin at concentrations ranging from 50 to 150 µM for 24 hours yielded promising results, notably leading to a reduction in the expression of Keap1, which is a negative regulator of Nrf2 [25]. This reduction potentially facilitates an increase in Nrf2 levels, thereby enhancing the cellular antioxidant response. Additionally, polydatin treatment significantly elevated the levels of glyoxalase-1, an enzyme critical for detoxifying methylglyoxal, while simultaneously decreasing the expression of the receptor for advanced glycation end products (RAGE) [25]. This suggests that polydatin may offer protective effects to RSC96 cells against methylglyoxal-induced toxicity, primarily by suppressing RAGE, which is known to mediate inflammatory responses associated with oxidative stress. Furthermore, treatment with polydatin led to a notable reduction in both the percentage of apoptotic cells and the production of ROS, indicating its potential role in promoting cell survival and maintaining cellular integrity under conditions of metabolic stress [25].

In an *in vivo* study involving diabetic rats, the administration of polydatin at doses of 25 mg/kg and 50 mg/kg over a two-week period resulted in significant improvements in both tail and paw withdrawal thresholds, indicating a restoration of nerve function deficits commonly associated with chronic diabetes [24]. Notably, the higher dose of 50 mg/kg led to a marked increase in the expression of key antioxidant enzymes, specifically NAD(P)H NQO1 and SOD2, in the sciatic nerve lysates when compared with diabetic control groups. This suggests that polydatin enhances not only antioxidant defences but also mitigates oxidative stress-related damage in peripheral nerves. Furthermore, polydatin treatment activated Sirt1, a critical regulator in cellular stress responses, which subsequently promoted mitochondriogenesis in the peripheral nerves of STZ-induced diabetic rats [24]. This activation played a pivotal role in reversing mitochondrial dysfunction by restoring the expression levels of essential proteins such as Sirt1, peroxisome proliferator-activated receptor gamma coactivator 1-alpha, Nrf2, and mitochondrial transcription factor A.

Moreover, polydatin demonstrated remarkable efficacy in facilitating the morphological restoration of damaged sciatic nerves in diabetic rats, leading to the development of more organized and structurally coherent nerve architectures [25]. The treated group exhibited a significant increase in the density of myelinated axons, which are crucial for efficient nerve signal transmission. Notably, the postcrush analysis revealed that the average length of myelinated axons in the polydatin-treated group exceeded 8 µm, in stark contrast with the shorter lengths of 4–6 µm observed in the nontreated group. This enhanced regeneration indicates not only a recovery from initial nerve injury but also an improvement in overall nerve functionality, suggesting that polydatin may play a vital role in promoting nerve health and recovery in diabetic neuropathy.

These findings suggest that polydatin is not only effective in healing diabetic nerve damage but also holds promise as a valuable therapy for diabetic neuropathy. By demonstrating the capacity to restore nerve function and improve nerve morphology, polydatin emerges as a potential candidate for enhancing treatment outcomes in individuals suffering from diabetic complications. However, to fully harness its therapeutic potential, further research is imperative to elucidate the specific neuroprotective mechanisms through which polydatin exerts its beneficial effects. Understanding how polydatin affects oxidative stress, apoptosis, and inflammation will be crucial, particularly regarding its influence on mitochondrial dynamics and the modulation of ROS production. Such insights could pave the way for optimizing polydatin’s application in clinical settings, ultimately leading to more effective management strategies for diabetic neuropathy and its associated challenges.

## Cardioprotective role of polydatin

Diabetic cardiomyopathy is a serious and progressive cardiovascular complication that arises from diabetes mellitus, characterized by a cascade of pathological changes that begin with cardiac fibrosis [57]. This initial stage can evolve into more severe forms of myocardial dysfunction, manifesting as both diastolic and systolic dysfunction, which can ultimately culminate in heart failure, significantly impacting patient morbidity and mortality [58]. One of the key contributors to the development of diabetic cardiomyopathy is inadequate autophagy, a critical cellular process responsible for maintaining cellular homeostasis and removing damaged organelles, which can exacerbate cardiac dysfunction when impaired [59]. Furthermore, the deficiency of Sirt3, a mitochondrial deacetylase, has been implicated in the deterioration of mitochondrial function, leading to contractile failure in cardiac tissues [60].

Research indicates that polydatin can effectively mitigate myocardial dysfunction in diabetic mice, highlighting its potential as a therapeutic agent for diabetic cardiomyopathy [61]. Specifically, treatment with polydatin has been shown to restore defective mitochondrial ultrastructure, which is often compromised in diabetic conditions. Additionally, polydatin significantly increases the number of autophagosomes in diabetic hearts, enhancing autophagic activity [61]. This protective effect is mediated through the activation of Sirt3, a key regulator that enhances cardiomyocyte autophagy flux, thereby promoting cellular repair and maintaining mitochondrial integrity. Furthermore, polydatin treatment has demonstrated a remarkable improvement in MMP when compared with high glucose conditions, suggesting a restoration of mitochondrial function that is crucial for overall cardiac health [49].

Polydatin’s protective effects extend significantly to improving myocardial dysfunction in diabetic rats, as evidenced by notable increases in left ventricular shortening fraction and ejection fraction, alongside reductions in cardiac hypertrophy and interstitial fibrosis [26]. In addition, polydatin pretreatment has been shown to mitigate high glucose-induced damage in cultured embryonic rat cardiac H9c2 cells in a dose-dependent manner, reinforcing its therapeutic potential [26]. This treatment effectively inhibited the increases in ROS, NADPH oxidase activity, and inflammatory cytokines observed in both *in vitro* and *in vivo* diabetic models. Furthermore, polydatin demonstrated its capacity to block the elevated expression of Nox2 and Nox4, as well as the NF-κB pathway in high glucose-stimulated H9c2 cells and diabetic hearts [26].

Oxidative stress is a crucial factor in the pathophysiology of myocardial ischemia and reperfusion (MI/R) injury, leading to significant cardiac damage and impaired function [62]. A recent study demonstrated that polydatin therapy can effectively mitigate myocardial oxidative stress under MI/R conditions by lowering levels of superoxide, reducing the expression of Nox2, and decreasing MDA levels, a marker of lipid peroxidation [27]. Concurrently, polydatin enhances the activity of myocardial SOD, an important antioxidant enzyme that protects cells from oxidative damage [51]. Furthermore, polydatin exhibits notable antinitrative effects during MI/R injury by suppressing the expression of inducible nitric oxide synthase (iNOS), which is responsible for the overproduction of nitric oxide (NO) that can exacerbate oxidative stress and contribute to cellular injury [27].

Polydatin’s cardioprotective effects in diabetic MI/R injury are significantly linked to the activation of the myocardial Notch1/hairy and enhancer of split-1 (Hes1) signalling pathway, which plays a vital role in cellular survival and differentiation during stress conditions [27]. This pathway is crucial for enhancing the heart’s resilience against ischemic injury, contributing to improved recovery and function. Additionally, the Akt signalling pathway is instrumental in mediating the antioxidative and antinitrative effects of polydatin within the context of diabetic MI/R damage [27]. By activating Akt, polydatin promotes cellular survival mechanisms and enhances the heart’s ability to cope with oxidative and nitrative stress, thereby reducing the extent of injury.

Compared with diabetic control groups, polydatin treatment demonstrated significant protective effects on heart function, as evidenced by increased left ventricular systolic pressure and a marked reduction in myocardial apoptosis [27]. This protective effect was quantified through various biomarkers, including a lower proportion of terminal deoxynucleotidyl transferase dUTP nick end labeling-positive nuclei, indicating reduced apoptotic cell death, as well as decreased expression of cleaved caspase-3 and diminished caspase-3 activity. Furthermore, in diabetic animals, levels of biomarkers associated with myocardial infarction, such as creatine kinase and creatine kinase-MB, were significantly elevated in the serum; however, polydatin effectively alleviated this increase, suggesting a restorative effect on myocardial integrity [27].

Polydatin demonstrates significant cardioprotective effects against hyperglycemia-induced myocardial damage in diabetic cardiomyopathy, making it a potential therapeutic candidate for this condition. Its ability to enhance autophagy, reduce oxidative stress, and improve overall cardiac function highlights its multifaceted role in mitigating the adverse effects of diabetes on the heart. By promoting autophagy, polydatin helps in the clearance of damaged cellular components, which is crucial for maintaining cardiomyocyte health. Additionally, its antioxidative properties help to counteract the detrimental effects of ROS, further preserving myocardial integrity. Collectively, these actions not only protect against myocardial injury but also support the restoration of cardiac performance in diabetic settings. However, further studies are warranted to elucidate the precise molecular mechanisms underlying these protective effects, as a deeper understanding could enhance the development of targeted therapies for managing diabetic heart complications.

## Impact of polydatin on endothelial health and vascular function

Endothelial cell dysfunction is a pivotal process implicated in various diseases, particularly diabetes mellitus, where it significantly contributes to the onset and progression of vascular complications [63]. In the context of diabetes, hyperglycaemia-induced endothelial dysfunction emerges as a common pathological basis for a range of vascular disorders, impacting overall cardiovascular health [64]. Factors such as excessive cytokines, inflammatory agents, and oxidative stress responses can adversely affect the expression and activity of endothelial nitric oxide synthase (eNOS) and cyclooxygenase-2, leading to decreased bioavailability of NO and prostacyclin [28]. This reduction contributes to impaired endothelium-dependent relaxation, affecting proper blood flow. The disruption of these endothelial functions not only exacerbates vascular complications in diabetes but also increases the risk of associated conditions such as atherosclerosis and cardiovascular disease [64].

Research indicates that polydatin can effectively restore endothelial function that has been impaired by high glucose levels, which is particularly relevant in the context of diabetes and its associated vascular complications. In a study designed to assess endothelium-dependent relaxation, it was found that high glucose concentrations, specifically at 55 mM, significantly hindered acetylcholine-induced vasodilation, a critical process for maintaining vascular health [28]. However, when treated with polydatin at a concentration of 10 μM, the endothelial function was successfully restored to the negative control levels. Additionally, the damaged aortic intima resulting from high glucose exposure showed significant improvement in polydatin-treated groups in a concentration-dependent manner (1, 3, and 10 μM) [28]. These findings suggest that polydatin not only ameliorates the functional impairments caused by high glucose but also contributes to the structural integrity of the endothelium, offering promising therapeutic potential for managing endothelial dysfunction in diabetic patients.

The beneficial effects of polydatin on endothelial function are mediated by several interconnected mechanisms that collectively enhance vascular health, particularly in high-glucose environments. One key mechanism involves the increased expression and activity of eNOS, which plays a crucial role in the production of NO, a vital molecule for promoting vasodilation and maintaining endothelial integrity [28,29]. In contrast, polydatin treatment is associated with decreased levels of iNOS, an enzyme that, when overexpressed, can lead to excessive production of NO and contribute to inflammatory processes [28,29]. This balance between eNOS and iNOS results in a significant enhancement of NO release, which is essential for proper endothelium-dependent relaxation and overall vascular function. Furthermore, it is noteworthy that this beneficial process is likely mediated, at least in part, by the activation of the peroxisome proliferator-activated receptor (PPAR) signalling pathway [28]. PPARs are nuclear receptors that regulate gene expression involved in lipid metabolism and inflammation, and their activation has been shown to promote endothelial health [65]. By engaging the PPAR-NO signalling pathways, polydatin effectively restores endothelial function even in the challenging conditions posed by high glucose levels. Additionally, polydatin reduced the expression of inflammatory factors such as NOD-like receptor thermal protein domain associated protein 3, vascular cell adhesion molecule 1, and IL-1β in aortic rings and cultured human umbilical vein endothelial cells (HUVECs) exposed to high glucose conditions [29].

Methylglyoxal, a reactive metabolite of glucose, has been increasingly recognized as a significant contributor to vascular cell death, particularly in the context of diabetic complications [66]. This compound is formed during glycolysis and is known to induce cellular stress and apoptosis, thereby exacerbating vascular damage associated with diabetes. In a study conducted by Pang et al. [30], the protective role of polydatin against methylglyoxal-induced apoptosis was rigorously examined using HUVECs as a model [30]. The results revealed that exposure to methylglyoxal led to a significant increase in the number of apoptotic cells compared with the vehicle control, underscoring the detrimental impact of this metabolite on endothelial cell viability. However, when treated with polydatin at concentrations of 50 and 100 μM, there was a remarkable attenuation of this apoptotic response, as polydatin effectively prevented the increase in apoptotic cell numbers induced by methylglyoxal.

Further investigations into the protective effects of polydatin against methylglyoxal revealed several critical mechanisms at play. Specifically, it was found that methylglyoxal significantly elevated the expression of cleaved caspase-3, a key indicator of apoptosis, along with an increased Bax/Bcl-2 ratio, which reflects a shift towards pro-apoptotic signalling [30]. Notably, polydatin pre-treatment effectively reduced both cleaved caspase-3 levels and the Bax/Bcl-2 ratio, suggesting that polydatin not only mitigates apoptosis but also promotes a more favourable balance between pro- and antiapoptotic factors [30]. In addition to its antiapoptotic effects, polydatin also protects against methylglyoxal-induced oxidative damage by suppressing the generation of ROS, which are harmful by-products of cellular metabolism that can lead to further cellular injury [30]. This antioxidant action is complemented by the enhancement of key antioxidant enzymes, including CAT, SOD, and GPx, which collectively work to neutralize ROS and reduce oxidative stress within the cells. Furthermore, pretreatment with polydatin was associated with the preservation of mitochondrial integrity, as evidenced by the prevention of mitochondrial morphological changes typically induced by methylglyoxal exposure [30]. Importantly, polydatin also mitigated the impairment of MMP, a critical indicator of mitochondrial function and health [29,30]. Additionally, it inhibited the dephosphorylation of Akt, a signalling pathway involved in cell survival and metabolism, which is often disrupted in the presence of oxidative stress and apoptosis [30].

A recent study involving HUVECs treated with high glucose has shown that polydatin effectively reduces pyroptosis, a form of programmed cell death characterized by inflammatory responses [29]. Pyroptosis is often associated with mitochondrial dysfunction, which is mediated by the excessive fission of mitochondria [67]. In these cells, overexpression of Drp1 led to extensive mitochondrial fission and the accumulation of ROS, both of which are key indicators of cellular stress and play a critical role in triggering inflammatory pathways that drive pyroptosis [29]. In particular, ROS accumulation is a major factor in the activation of caspase-1, an enzyme central to pyroptosis [68]. Polydatin exerts its protective effects by modulating mitochondrial dynamics. Specifically, it reduces the expression of Drp1, a protein involved in mitochondrial fission, decreases ROS and caspase-1 levels, and enhances the p-Drp1/Drp1 ratio [29]. This alteration in Drp1 phosphorylation suggests a shift towards maintaining mitochondrial integrity, reducing mitochondrial fragmentation, and minimizing oxidative stress, ultimately protecting against the activation of caspase-1 and the progression of pyroptosis.

The above findings suggest that polydatin has significant protective effects against diabetic vascular complications by restoring endothelial function and preventing apoptosis in endothelial cells. These effects are particularly crucial, as endothelial dysfunction is a hallmark of vascular complications associated with diabetes, contributing to issues such as impaired blood flow, increased vascular permeability, and heightened inflammation [64]. By effectively inhibiting oxidative stress, which plays a central role in endothelial cell injury, polydatin helps mitigate the harmful effects of ROS that can lead to cell death and further exacerbate vascular damage [30]. Moreover, polydatin’s ability to maintain mitochondrial function is particularly noteworthy, as mitochondria are essential for energy production and metabolic regulation in endothelial cells [30]. When mitochondrial health is compromised, it can trigger a cascade of detrimental events, including increased oxidative stress, apoptosis, and pyroptosis [69]. By preserving mitochondrial integrity and function, polydatin not only supports cell survival but also promotes overall vascular health, thus contributing to improved endothelial function.

## Conclusions

**Figure 3:**
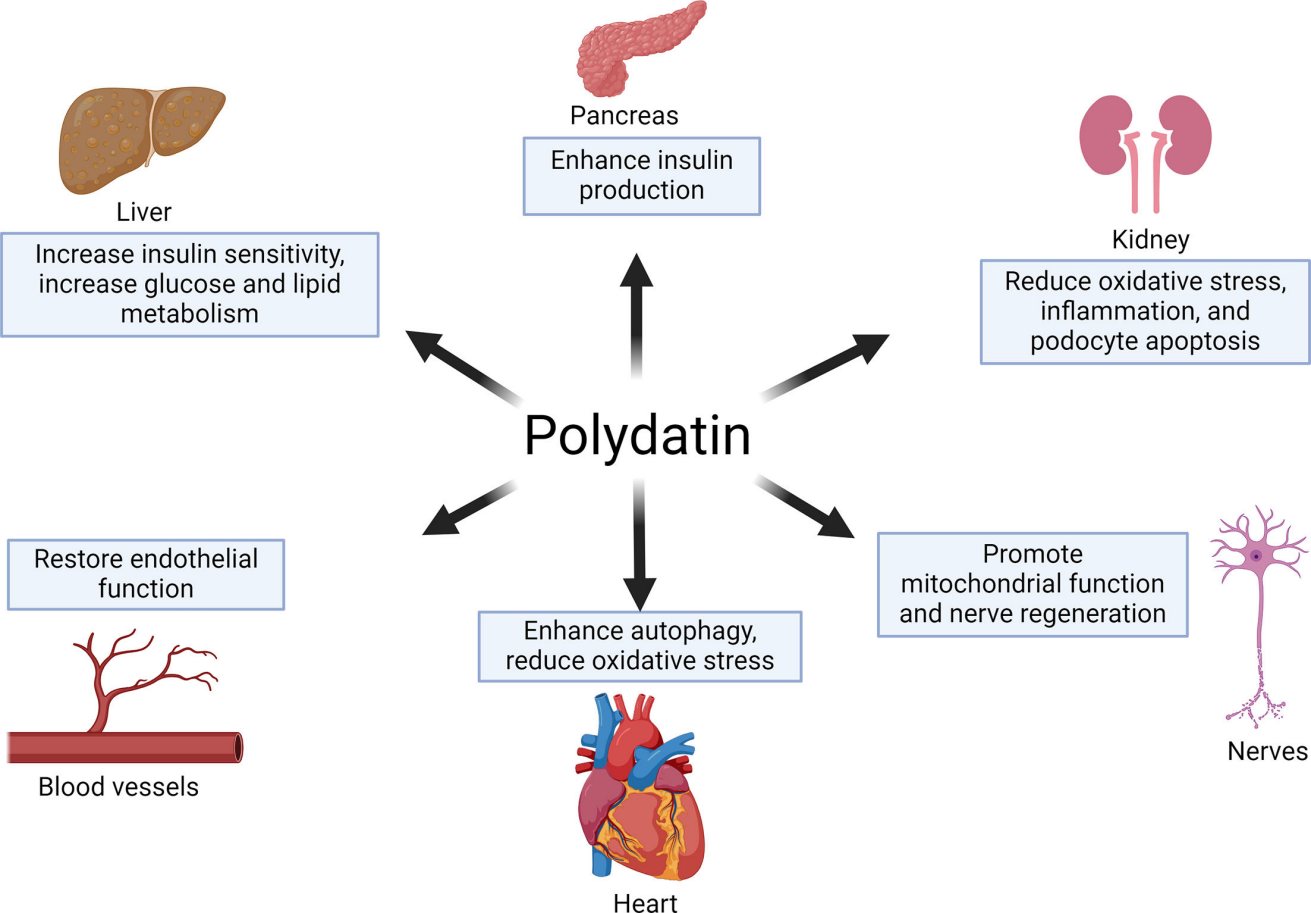
Protective effects of polydatin against a wide range of diabetic complications. Created in BioRender. Tang, K. (2025) https://BioRender.com/f61o708.

Overall, the evidence supports the protective effects of polydatin in the management of diabetes mellitus and its related complications (Figure 3). This comprehensive review has highlighted polydatin’s multifaceted roles in enhancing insulin sensitivity, improving pancreatic function, and mitigating the risk of metabolic syndrome. Its ability to regulate blood glucose levels and combat oxidative stress positions polydatin as a potential therapy for diabetes management. Moreover, polydatin demonstrates significant protective effects against various diabetes-related complications, including nephropathy, neuropathy, cardiomyopathy, and vascular disorders. The compound’s mechanisms of action encompass the activation of crucial signalling pathways, such as Nrf2 and Sirt1, the modulation of inflammatory responses, and the restoration of mitochondrial function. These actions collectively contribute to its protective effects on renal, neural, cardiac, and vascular health.

Although showing a wide range of potential pharmacological benefits, concerns have been raised regarding the toxicity of polydatin, especially at high doses. However, comprehensive data on its safety and toxicity are still lacking. As such, further research is needed to establish safe dosage guidelines for human consumption and to better understand the potential risks associated with its use, while taking necessary precautions to prevent adverse effects. A recent study by Schimith et al. [70] assessed the toxicity of polydatin using the zebrafish model, evaluating survival, morphology, hatchability, cardiac function, and behaviour [70]. The findings suggest that polydatin exhibits a promising nontoxic profile up to 435 μM, supporting its potential for human therapeutic applications and consumer products, and encouraging further research and clinical investigation.

Despite the encouraging findings from animal models and *in vitro* studies, there remains a critical need for additional research to comprehensively understand the therapeutic potential of polydatin in human populations. Specifically, well-controlled clinical trials are essential to evaluate its safety, efficacy, and long-term effects in diverse patient populations. Clinical data are crucial to confirm whether the observed benefits in preclinical models translate into meaningful outcomes in humans. If validated through rigorous clinical evidence, polydatin could represent a valuable natural treatment strategy for managing diabetes and its complications, offering a complementary strategy alongside conventional therapies. Such an approach would not only target the metabolic disturbances associated with diabetes but also promote a more holistic, patient-centred model of care that focuses on enhancing overall health, minimizing side effects, and improving the quality of life for individuals living with diabetes.

## Abbreviations

AGEs: advanced glycation end products
AMPK: AMP-activated protein kinase
ARE: antioxidant response element
CAT: catalase
CKIP-1: casein kinase 2 interacting protein 1
Cx32: connexin 32
GCK: glucokinase
GMCs: glomerular mesangial cells
GPx: glutathione peroxidase
HES1: hairy and enhancer of split‑1
HO-1: heme oxygenase-1
ICAM-1: intercellular adhesion molecule-1
IL-1β: interleukin-1 beta
Keap1: Kelch-like ECH associated-protein 1
LDL-C: low-density lipoprotein cholesterol
LDLR: low-density lipoprotein receptor
MDA: malondialdehyde
MI/R: myocardial ischemia and reperfusion
MMP: mitochondrial membrane potential
NF-κB: nuclear factor kappa B
NO: nitric oxide
Nox: NADPH oxidase
Nrf2: nuclear factor E2-related factor 2
PCSK9: proprotein convertase subtilisin/Kexin type 9
PPAR: peroxisome proliferator-activated receptor
RAGE: receptor for advanced glycation end products
ROS: reactive oxygen species
SOD: superoxide dismutase
S1P: sphingosine 1-phosphate
STZ: streptozotocin
Sirt: sirtuin
SphK: sphingosine kinase
TGF-β: transforming growth factor beta
TNF-α: tumour necrosis factor alpha
eNOS: endothelial nitric oxide synthase
iNOS: inducible nitric oxide synthase
